# Phytochemical Investigation and Therapeutical Potential of *Cotinus coggygria* Scop. in Alloxan-Induced Diabetic Mice

**DOI:** 10.1155/2022/8802178

**Published:** 2022-12-31

**Authors:** Shahid Rahman, Gul Jan, Farzana Gul Jan, Hafeez Ur Rahim

**Affiliations:** ^1^Pharmacology Lab, Department of Botany, Abdul Wali Khan University Mardan, Khyber Pakhtunkhwa, Pakistan; ^2^Department of Soil and Environmental Sciences, The University of Agriculture Peshawar, Pakistan

## Abstract

Plants are a significant source for the development of new phytomedicines due to their great clinical benefits, efficiency, cost-effectiveness, fewer side effects, and more affordable therapies. Numerous plants used in traditional treatments, such as *Cotinus coggygria* Scop., have been effective in the treatment of diabetes mellitus (DM). Therefore, the study is aimed at assessing the phytochemical, antioxidant, and antidiabetic properties of *C. coggygria*. The hypoglycemic and hypolipidemic activity was evaluated in Swiss male Albino mice by administering an oral dose of 150-250 mg/kg of *C. coggygria* extracts in alloxan-induced diabetic mice for 15 days. The antioxidant activity and phytochemical composition of the extracts were assessed by using *α*, *α* diphenyl-*β*-picrylhydrazyl (DPPH) and hydrogen peroxide scavenging assays and through standard chemical procedures. The effects of extracts on blood glucose, body weight, lipid profile, and biochemical parameters like total cholesterol (TC), triglyceride (TG), low-density lipids (LDL), high-density lipids (HDL), plasma insulin, liver glycogen, aspartate aminotransferase (AST), alanine aminotransferase (ALT), alkaline phosphatase (ALP), urea, and creatinine were determined according to standard procedures. The activities of antioxidant enzymes such as superoxide-dismutase (SOD), peroxidase (POD), and catalase (CAT) were also analyzed spectrophotometrically. The hypoglycemic and hypolipidemic effects with chloroform extracts of 250 mg/kg were found significant in the treatment of diabetes in alloxanised mice compared to the diabetic group. The haematological parameters such as TC, TG, HDL, LDL, creatinine, urea, AST, ALT, and ALP were significantly improved (*p* < 0.01) by the chloroform extract of 250 mg/kg compared to the diabetic group. Treatment for 15 days showed significant elevation (*p* < 0.01) of antioxidant enzymes. Fourier-transform infrared spectroscopic (FTIR) and gas chromatography–mass spectrometry (GC-MS), column chromatography (CC), and nuclear magnetic resonance (NMR) analyses tentatively identified different phytoconstitutents and metabolites in *C. coggygria* leaves, which have been reported to possess antihyperglycemic properties. In conclusion, the chloroform extract of 250 mg/kg of *C. coggygria* possesses significant hypoglycemic and hypolipidemic potential which may prove the claimed use of the plant in amelioration of diabetes and associated complications in folkloric medicine. Additional studies are required for the purification, characterization, and structural elucidation of bioactive compounds.

## 1. Introduction

DM is a serious and incurable metabolic disease defined by high blood glucose levels produced by a relative or absolute shortage of insulin or insulin's inability to work on its target tissues [1]. Insulin insufficiency is known to result in hyperlipidemia and fatty liver by promoting lipolysis in adipose tissue. DM associated with hyperlipidemia involves an irregular circulating lipid profile including low levels of HDL, elevated levels of LDL, and TG [2]. Diabetic complications include increased ketogenesis, gluconeogenesis, and increased risk of coronary heart diseases [3].

The currently available therapies as treatments for DM include biguanides, sulfonylureas, and thiazolidinediones, among other synthetic medications [4]. Although promising for diabetes management, each of the aforementioned oral antidiabetic medications has side effects that have been documented in the literature. In this context, medicinal plants are used as supplements to current medicines for treating DM because they are thought to be reliable sources of hypoglycemic compounds [5]. Plant extracts can lower blood sugar levels in a number of ways, including by containing compounds that act like insulin, blocking the action of insulin, and promoting pancreatic beta-cell regeneration [6]. A quarter of all medications administered worldwide come from plants that are already used to make conventional medicines [7]. Among many indigenous plants, *C. coggygria* is used in the treatment of DM in many traditional herbal medicines.

*C. coggygria* Scop. (syn. *Rhus cotinus* L.) is a deciduous and slow-growing shrub of the family Anacardiaceae. The family is distributed in the tropics of Asia, Africa, and America with some species occurring in subtropical and temperate areas [8]. The leaves of the plant are consumed as an infusion in Turkish folklore medicine for its anti-inflammatory, hepatoprotective, cytotoxic, antioxidant, antiseptic, antihemorrhagic, wound healing, antiviral, and antimicrobial properties both *in vitro* and *in vivo* [9]. The leaves of the plant are also used as antidiarrhetic, gastric and duodenal ulcer, and paradontosis in Bulgarian phytotherapy [10]. According to ethnopharmacological investigations, the plant has long been utilized in traditional herbal treatments for the management of DM due to its considerable hypoglycemic effect with concomitant improvement in the antioxidant status [9]. The leaf extract of *C. coggygria* with other extracts is effective in preventing and reducing the risk as well as minimizing the symptoms of hemorrhoids in Chinese traditional medicines [11]. The *C. coggygria* dried leaf and twigs are used to eliminate dampness and heat and as an antipyretic [12]. Similarly, numerous phytocompounds have been discovered and isolated from various parts of *C. coggygria*. According to the reports, the primary group of physiologically active compounds in the methanolic and ethyl acetate extracts of various parts of *C. coggygria* includes total flavonoids, phenols, and tannins [13–15].

The most valuable secondary metabolites found in *C. coggygria* that have anti-inflammatory, astringent, and antiseptic properties are tannins, the content of which varies from 6 to 30%, depending on the time of harvest and the quantity of sunlight absorbed by the leaf. The root-based decoction from *C. coggygria*, for instance, has antipyretic effects and is also used to treat pharyngitis and stomatitis. Similarly, bactericidal activities can also be found in aqueous and alcoholic extracts of the plant. It is also worth noting that the *C. coggygria* leaves are included in the pharmacopoeia because of its medicinal importance. Additionally, the extracts of *C. coggygria* also exhibit excellent antioxidant activity in response to DPPH and were able to partially or fully block the peroxidation of plant lipids. Gallic acid, quercetin, butin, myricetin, and disulfuretin are identified in various portions of the plant-based on high-performance liquid chromatography (HPLC) profiles and other analysis on *C. coggygria* using different solvents [16]. Following phenolic acids, sterols, and other secondary metabolites, the flavonoids are the most significant and prolific category of biologically active constituents of *Cotinus* species [17].

Various extracts of *C. coggygria*, including n-hexane, chloroform, ethyl acetate, and aqueous fractions, were subjected to phytochemical studies which revealed the presence of phenolic compounds, flavonoids, cardiac glycosides, alkaloids, terpenoids, tannins, and saponins as well as a rich source of antioxidants [18]. According to the literature, very limited study has been conducted so far to evaluate the *C. coggygria* potential for antidiabetic action and its corresponding biological characteristics. Due to the plant's active constituents and having high hypoglycemic potential in herbal recipes, traditional health practitioners also recommend it to treat diabetes. Therefore, the current research sought to examine and validate the antidiabetic and antihyperlipidemic properties of *C. coggygria* plant extracts as well as to explore the phytochemical components through various phytochemical tests and characterizations.

## 2. Materials and Methods

### 2.1. Collection of Plant Materials

The fresh leaves of *C. coggygria* were collected during the month of June 2019 from the woody habitat of Chamla Mountains of District Buner, Khyber Pakhtunkhwa, Pakistan, after approval of the study from the Bioethics Committee of the Faculty of Biological Sciences, Abdul Wali Khan University Mardan. The taxonomic identity and authentication of the desired plant were determined by the herbarium unit of the Department of Botany, Abdul Wali Khan University Mardan, and a voucher specimen no. Bot. 201904 (AWK) was deposited.

### 2.2. Preparation of Extracts for Chemical and Bioactive Studies

The fresh C. coggygria leaves were examined closely, and any degraded sections or foreign particles were removed and properly washed with tap water. The leaves were mechanically milled into fine powder after being shade dried for roughly two weeks in a well-ventilated area. According to the directions in the literature, the powdered plant was weighed (1 kg), and the crude methanolic extract (410 gm) was prepared [19]. The plant's metabolite content was extracted using aqueous methanol (80%) by adopting the cold maceration method.

### 2.3. Phytochemical Analysis

#### 2.3.1. Qualitative Phytochemical Screening

The preliminary phytochemical screening tests were conducted for determining the secondary metabolites in the plant such as phenols (ferric chloride test) [20], alkaloids (Mayer's test) [21], glycosides (Keller-Killiani's test) [22], reducing sugars (Fehling's test) [23], saponins (frothing test) [24], flavonoids (Shinoda's test) [25], tannins (ferric chloride test) [25], steroids (emulsion test) [26], proteins (xanthoproteic test) [22], terpenoids, quinones and anthraquinones (Borntrager's test) [27], coumarins (NaOH solution test) [28], terpenoids (Salkowski's test) [21], and phlobatannin (HCL test) [29].

#### 2.3.2. Quantitative Analysis of Phytochemicals

The quantitative determination of phytochemicals in the methanolic extract and fractions was carried out spectrophotometrically for phenolic, flavonoid, tannin, sugar, and protein contents by the “Folin-Ciocalteu method” [30], aluminum chloride assay [31], phenol sulphuric acid method [32], and Lowry's method [33] at wavelengths of 765 nm, 510 nm, 700 nm, 540 nm, and 660 nm, respectively.

### 2.4. Antioxidant Study

The antioxidant potential of extracts and various fractions of *C. coggygria* were determined by using DPPH and hydrogen peroxide assay methods [34]. The decrease of the absorption at 517 nm of the DPPH solution after the addition of the antioxidant was measured in a cuvette containing 2.96 ml of ethanolic DPPH (0.1 mm) solution and 20 to 200 *μ*g/ml of plant extract. The setup was kept in dark at room temperature, and the absorption was monitored after 20 minutes. The chromatographic method was optimized, according to reaction time (slow, rapid kinetics) and the linearity range of DPPH radical depending on the detection conditions as well as the kind of investigated antioxidants (reference chemicals and the ground elder prepared from fresh and dry plants). The scavenging capacity was expressed by the use of the percentage of peak inhibition and the IC_50_ parameters.

(1) $$\begin{array}{c} \text{Scavenging }\left(\%\right)=\frac{\left(A_{\text{control}}-A_{\text{sample}}\right)}{A_{\text{control}}}\times 100. \end{array}$$

Similarly, the ability of the extract to scavenge hydrogen peroxide was determined by transferring the aliquot of 0.1 ml of extracts into the Eppendorf tubes, and their volume was made up to 0.4 mM phosphate buffer followed by the addition of 0.6 ml of H_2_O_2_ solution. The reaction mixture was vortexed, and after 10 min of reaction time, the absorbance was measured at 230 nm. Ascorbic acid was used as a reference compound.

### 2.5. Experimental Animals and Treatments

The male Swiss albino mice, aged 3-5 weeks and weighing 25-30 g, were acquired from the Veterinary Research Institute (VIR), Peshawar, Pakistan. The mice were kept in neat stainless-steel cages and given commercial stock chow and water ad libitum for a week under optimal laboratory conditions of a narrowly sustained temperature of 25 ± 2°C. The experimental animal procedures were carried out with the approval of the ethical committee of the Abdul Wali Khan University Mardan (0014/2019).

### 2.6. Toxicity Evaluation in Mice

Based on OECD standards 2008:425 [35], initial toxicological investigations were carried out to determine whether the plant extracts had any hazardous effects. The overnight-fasted mice were divided into groups and given extracts at progressively higher doses (500, 1000, 2000, and 2500 mg/kg). The animals were observed for any gross behavioral indications, such as tremors, sleepiness, piloerection, writhing, sluggishness, restlessness, convulsions, weight loss, and paralysis, at regular intervals [35].

### 2.7. Diabetes Induction

Experimental diabetes was induced in mice by a single intraperitoneal (IP) injection of alloxan monohydrate (150 mg/kg) dissolved in sterile normal saline following the standard protocol [36]. The animals were given feed ad libitum and 5% dextrose solution after 30 min of alloxan administration to overcome the early hypoglycemic crisis. The mice whose blood glucose level was more than 200 mg/dl and showed signs of polyuria, polyphagia, and polydipsia were considered as diabetic and selected for subsequent study [37]. The treatment with methanolic extracts, glibenclamide, and solvent fractions was initiated 72 h after alloxan injection into the mice.

### 2.8. Experimental Protocol and Procedure

48 experimental mice were divided into eight groups of six mice each to assess the antidiabetic activity. Group 1 was normal saline-treated only with Tween 80, group 2 was diabetic control received only alloxan 150 mg/kg, and group 3 was diabetic mice treated with glibenclamide (10 mg/kg) [38]. Group 4 had diabetic mice treated with the crude methanolic extracts of 250 mg/kg, and groups 5, 6, 7, and 8 included alloxan-induced diabetic mice provided with n-hexane, chloroform, ethyl acetate, and aqueous extracts, respectively, at a concentration of 250 mg/kg [39]. The extracts and saline solutions were administered orally via oral gavage. The dose of 250 mg/kg body weight was selected based on the effectiveness of their traditional claim.

### 2.9. Determination of Blood Glucose

The blood glucose level was checked for each group by collecting blood samples from the overnight-fasted mice tail vein by using oxidase-peroxidase reactive strips and a glucometer (Accu-ChekActive, Roche Diagnostics, Germany) ([40]. The tails were then rubbed with ethanol to avoid infection. The fluctuations in the weight of the animals were also monitored and recorded on the same days.

### 2.10. Collection of Blood Samples

The blood samples for the serum insulin and lipid profiles were obtained by cardiac puncture from overnight-fasted mice under diethyl ether anaesthesia and set aside for 30 minutes to clot. The serum was centrifuged for separation at 3000 rounds per minute, for 10 min at 25°C, and was used for different biochemical measurements [40].

### 2.11. Determination of Metabolic Parameters

The serum collected was analyzed subsequently for TC, TG, HDL, LDL, ALP, serum creatinine, and urea, respectively, by the methods of Friedewald et al. [41]. The AST and ALT activities were determined according to the method of Reitman and Frankel [42], by using commercial diagnostic kits (BT, 2000 plus, Germany).

### 2.12. Pathological Histology

The whole animals were sacrificed by the cervical dislocation at the end of the stipulated period (15 days), and the liver, pancreas, and kidney were collected from all groups, immediately excised, washed with chilled isotonic saline, and stored at -80°C until analyzed for histopathological evaluation by the paraffin method. The different histopathological parameters observed were cytoplasmic degeneration, Kupffer's cell activation, necrotic foci, hemorrhage, and also infiltration of inflammatory cells. Similarly, kidney histopathology considered the exposure of GNPs to pathological changes in the form of diminished and distorted glomeruli, edema exudate, dilated tubules, mild necrosis, and cellular degeneration. The tissue segments were primed with a microtome and stained with hematoxylin and eosin dyes so as to be observed under a light microscope [43].

### 2.13. In Vitro Membrane Stabilizing Activity

The membrane-stabilizing effects of the extracts were tested on human erythrocytes at a wavelength of 560 nm [44] to predict the in vitro antidiabetic activity of the plant.

### 2.14. Measurement of Antioxidant Enzyme Activity

The SOD, POD, and CAT assays were carried out to spectrophotometrically at 25°C by monitoring the absorbance at 420 nm, 560 nm, and 240 nm, respectively [45].

### 2.15. FTIR Analysis

The FTIR spectrum was used to detect the characteristic organic groups specially the alcoholic group in the powdered extract of *C. coggygria* in the frequency range of 4000–500 cm^−1^ and was recorded from KBr pellet in FTIR spectroscopy [46].

### 2.16. XRD Analysis

X-ray diffraction (XRD) analysis was carried to know the crystalline or amorphous nature of the extract of C. coggygria.

### 2.17. GC-MS Analysis of the Extract

GC-MS analysis of the chloroform extract was performed on a PerkinElmer Clarus 600 GC System fitted with a Rtx-5MS capillary column (30 m × 0.25 mm internal diameter, 0.25 *μ*m film thickness, and maximum temperature 350°C), coupled to a PerkinElmer Clarus 600C MS. Ultra-high-purity helium (99.99%) was used as carrier gas at a constant flow rate of 1.0 ml/min. The injection, transfer line, and ion source temperatures were all 290°C. The ionizing energy was 70 eV. Electron multiplier voltage was obtained from autotune. The oven temperature was programmed from 60°C (hold for 2 min) to 280°C at the rate of 3°C/min [47].

### 2.18. Identification of Phytocompounds

The National Institute of Standards and Technology's 2008 (NIST-2008) database, which contains over 62,000 patterns, was used to interpret GC-MS mass spectra based on a comparison of known and unknown spectral components in the NIST library.

### 2.19. Column Chromatography

CC was done on the silica gel (0.063-0.200 mm, 70-230 mesh ASTM, MERCK). TLC (thin layer chromatography) was performed on the precoated silica gel (GF254, 0.5 mm thick, 20 × 20 cm, MERCK) plates. The purity of the isolated compounds was checked on the TLC plates with various solvent systems using n-hexane, methanol, acetone, and chloroform giving a single spot. The chloroform soluble fraction (148 g) was subjected to a silica gel column (70-230 mesh) CC using as solvent system a gradient of n-hexane and CHCl_3_ (n-hexane:CHCl_3_, 88 : 12, 85 : 15, 80 : 20, 78 : 22, 75 : 25, 70 : 30, 65 : 35, 60 : 40, 20 : 80, 25 : 75, 0 : 100) (5000 ml for each gradient) and followed by acetone up to 100%. Eight fractions (fractions 1-8) were collected, having the elution volumes as 3550 ml, 4500 ml, 3250 ml, 3350 ml, 3650 ml, 4000 ml, 27050 ml, and 3200 ml, respectively.

### 2.20. Identification of Compounds

Using 1H and 13C NMR spectroscopy, all isolated chemicals were identified. The sample's NMR analysis was carried out with some modifications from Wijaya et al. (2017)[48]. A sample of 50 mg was diluted with CD3OD, vortexed for 2 minutes at room temperature, and then subjected to 15 minutes of ultrasonication at 1000 g. A 500 MHz NMR (JEOL NMR Spectrometer, USA) was used to analyze the eight hundred microliters of samples, with deuterated methanol acting as the internal lock. The frequency at which the NMR spectra were captured was 500.16 MHz. Each 1H NMR spectrum required 128 scans, 10 min, 26 s of acquisition time, and 1.5 s of relaxation delay time. The temperature was kept at 25°C. The TSP was used as a reference at *δ* 0.00.

Phasing, baseline, and reference corrections of NMR spectra were performed manually using MNOVA version 13.0. The metabolites were identified by comparing the ^1^H NMR spectra of the sample and the published literature.

### 2.21. Statistical Analysis

The data were analyzed by using IBM (SPSS V-20, IBM Corp., Armonk, N.Y., USA). The sampling units were 8, and each treatment (diabetic control, normal saline, glibenclamide, methanolic extract, n-hexane, chloroform, ethyl acetate, and aqueous fraction) was replicated five times, i.e., day 0, day 4, day 7, day 11, and day 15. The dependent variables were blood glucose level, body weight, lipid profile, biochemical parameters, and antioxidant enzymes while the treatments were considered as independent variables. All the data were expressed as mean ± standard error of mean (SEM), and the statistical investigations were carried out using one-way analysis of variance (ANOVA) followed by the Dunnet postcomparison test. The variations were considered significant at *p* < 0.05.

## 3. Results

### 3.1. Extraction Yield

The percent extractive yields of each fraction (methanolic, n-hexane, chloroform, ethyl acetate, and aqueous) obtained were 88%, 11.6%, 33%, 10.8%, and 30.8%, respectively.

### 3.2. Phytochemical Analysis

#### 3.2.1. Qualitative Phytochemical Evaluation

*C. coggygria* plant showed the existence of alkaloid, flavonoid, sugar, protein, glycosides, saponins, tannin, phytosterol, phenol, quinones, terpenes, fats, phlobatanin, and coumarin.

#### 3.2.2. Quantitative Determination of Phytochemicals

The quantitative spectrophotometric analysis of phytochemicals indicated the presence of total phenols, flavonoids, tannins, sugar, and protein in the plant extracts of *C. coggygria* (Table 1).

### 3.3. In Vitro Antioxidant Assays

The methanolic and chloroform extracts of *C. coggygria* possess the strongest ability to scavenge against DPPH radical and hydrogen peroxide as compared to other extracts (Table 2). The percentage of inhibition was observed in both the antioxidant models that free radicals were scavenged by the plant extract in a concentration-dependent manner up to the given concentration (0.05 mg/ml, 0.08 mg/ml, and 0.1 mg/ml). The percentage inhibition of scavenging activities of the DPPH and H_2_O_2_ showed higher inhibition at 0.1 mg/ml concentrations.

### 3.4. Acute Toxicity Evaluation

The extracts were determined to be safe in mice up to a dose level of 2000 mg/kg body weight because they did not produce any obvious toxicity indications. Based on these findings, a 250 mg/kg dose was chosen as the maximum dose for further experimentation. This was supported by the fact that all mice were physically active for two weeks and showed no major changes in their sensory or motor abilities, weight loss, sluggishness, respiration, paralysis, convulsions, or mortality.

### 3.5. Effect of Extracts on Glucose Level in Alloxan-Induced Hyperglycemic Mice

Hyperglycemic mice treated with alloxan had higher blood glucose levels compared to normal mice. The chloroform extract (250 mg/kg) of *C. coggygria* improved the hypoglycemic effects significantly (*p* < 0.01) in alloxan-induced diabetic mice (Table 3).

### 3.6. Changes in Body Weight

The body weight of diabetic mice was significantly lower than that of normal mice, which was significantly (*p* < 0.05 and *p* < 0.01) improved by the crude methanolic extract and chloroform fraction at the conclusion of the 15-day treatment period.  Table 4 demonstrates that other extracts had no impact on body weight.

### 3.7. Antihyperlipidemic Effects

Induction of diabetes by the alloxan significantly increased serum TC, TG, and LDL and decreased serum HDL in diabetogenic mice as compared to normal mice. The orally administered chloroform extract (250 mg/kg) significantly reduced (*p* < 0.01) the levels of TC, TG, and LDL and significantly elevated (*p* < 0.01) the levels of HDL (Table 5).

### 3.8. Effects on Biochemical Parameters

The concentration and activity of ALT, AST, and ALP in alloxan-induced diabetic mice significantly increased (*p* < 0.01), indicating liver damage. Similar to this, diabetic mice showed a substantial rise (*p* < 0.01) in serum creatinine and urea concentrations, indicating the renal impairment. The *C. coggygria* chloroform extract markedly improved these parameters (Table 6).

### 3.9. Determination of Antioxidant Enzymes

The activities of CAT, SOD, and POD in normal and diabetic mice are shown in Table 7. In diabetic mice, the activities of CAT, SOD, and POD were significantly decreased (*p* < 0.05 and *p* < 0.01). The values were brought back to near normal after the administration of methanolic and chloroform extracts (250 mg/kg).

### 3.10. Effect of Extracts on Hypotonic Solution-Induced Haemolysis of Erythrocytes

This study demonstrated it that the chloroform extracts of *C. coggygria* (at conc. 1 mg/ml) were significantly potent on human erythrocytes compared to the standard acetylsalicylic acid, adequately protecting it against the hypotonic solution and heat-induced *in vitro* (Table 8).

### 3.11. FTIR Spectroscopic Analysis

**Figure 1:**
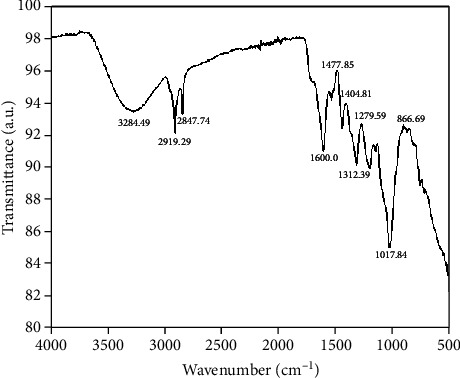
FTIR spectrum of *C. coggygria*.

Functional groups isolated from *C. coggygria* were identified from FTIR spectroscopic analysis. The FTIR data of the isolated compounds are shown in Figure 1 and Table 9.

### 3.12. Isolation of Compounds

The results of the isolation of individual biologically active substances from the leaves of *C. coggygria* chloroform extract by preparative chromatography are p-hydroxybenzoic acid, ursolic acid, betulonic acid, 5,2′,6′-trihydroxy-7-methoxyflavone, quercetin, 5,7,2′-trihydroxyflavone-2′-O-glucopyranoside, disulphuretin, sulphurein, myricetin, butein, sulphuretin, kaempferol, and derivatives of gallic and benzoic acids like (1) 7-*O*-*β*-D, (2) pentagalloyl glucose, (3) methyl gallate, (4) 3-*O*-*α*-L-rhamnofuranoside, and (5) —gallic acid.

### 3.13. Metabolite Profiling with NMR

**Figure 2:**
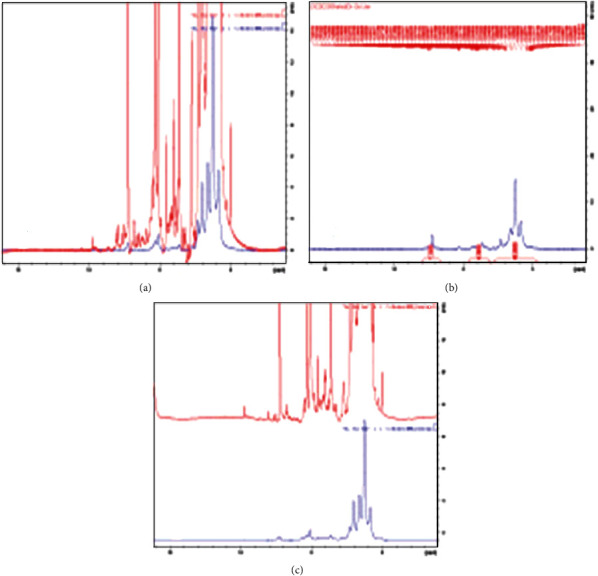
^1^H NMR typical spectrum (600 MHz, CD_3_OD:CDCl_3_) of *C. coggygria* chloroform extract sample in (a) high-, (b) middle-, and (c) low-frequency regions, showing the main metabolite classes associated with principal peaks.

^1^H NMR spectral of all samples showed high-intensity Hz in the region of *δ* 3.0-5.5, which could be attributed to saccharides/sugars (Figure 2(b)) present in all extracts especially *α*-glucose (*δ* 5.11-5.13, d; *J* = 3.8 Hz) and *β* glucose (*δ* 4.46-4.5, d; *J* = 7.8 Hz). Other high-intensity signals could also be assigned as amino acid signals including alanine (*δ* 1.45-1.48, d; *J* = 7.2 Hz), glutamate (*δ* 4 2.04-2.07, m), and threonine (*δ* 1.32, d; *J* = 6.6 Hz). In contrast, all spectra had lower intensity at 5.70–9.00 ppm regions, which varied between extracts. The signals in this region (Figure 2(a)) are typically for phenolic compounds, including flavonoids. The typical NMR signals identified in *C. coggygria* spectra (Figure 2(c)) after comparing with different NMR data included 4,5-di-O-caffeoylquinic acid, methyl ester, esculetin, myricetin, gallic acid, coumaric acid, syringic acid, *β*-sitosterol glucopyranoside, mupinensisone, and stigmasterol.

### 3.14. XRD Analysis

**Figure 3:**
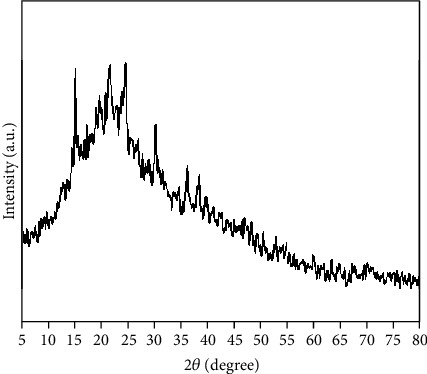
XRD pattern of *C. coggygria*.

XRD measurement was performed to validate the phase analysis of the methanolic extract of *C. coggygria*. No distinctive diffraction peaks have been observed in the whole spectrum, indicating the amorphous nature of the extract (Figure 3).

### 3.15. GC-MS Analysis

**Figure 4:**
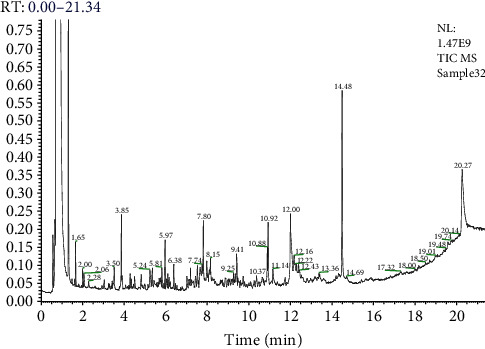
GC-MS analysis of chloroform extract of *C. coggygria*.

Based on the positive match of experimental mass spectra with the NIST MS database and data from the literature, the GC-MS study led to the preliminary identification of 36 chemicals in the chloroform extract of *C. coggygria* as shown in Table 10 and Figure 4.

### 3.16. Antidiabetic Compounds Identified from GC-MS

Table 11 displays the important antidiabetic compounds isolated from GC-MS analysis of chloroform fraction reported as having antihyperglycemic properties.

## 4. Discussion

DM is a noncommunicable disease often inherited but can sometimes be acquired as a result of lifestyle choices. There is no effective treatment technique or drug to cure diabetes in current medicine [60]. Antidiabetic medicinal plants can be a valuable resource for the development of safer, more affordable antidiabetic medications. *C. coggygria*, in this regard, has strong medicinal characteristics and is used in several regions of the world as an antidiabetic, antiseptic, anti-inflammatory, antihemorrhagic, and antibacterial, as well as for wound healing. In order to substantiate its traditional utilization in the treatment of DM in people, the current studies evaluated the phytochemical, antioxidant, antidiabetic, and antihyperlipidemic potential of *C. coggygria* in alloxan-induced diabetic mice. The plant extract is nontoxic and safe for use because no pathological abnormality or mortality was recorded in mice.

Plant extracts may include hypoglycemic substances that account for the drop in blood sugar levels observed in mice with transient hyperglycemia. These substances work by either causing the islets of the Langerhans cells to release more insulin or by blocking specific blood glucose-producing enzymes [61]. The mechanism of action would be similar to that of glibenclamide, which raises plasma insulin concentration by inducing insulin exocytosis in beta-cells. This has an insulin-modulating effect on peripheral tissues, such as the liver and skeletal muscle, and causes a rapid drop in mice's hyperglycemia [62]. Alloxan acts as diabetogenic by the destruction of the beta-cells of islets of Langerhans and causes a massive reduction in insulin release, thereby inducing hyperglycemia. Alloxan-induced diabetes in mice manifests a considerable loss of body weight, which is brought on by an increase in the degradation of structural proteins or muscle tissue [63]. However, when compared to diabetic control, treatments with orally administered methanolic and chloroform extracts significantly increased the body weight, indicating their protective effect in preventing muscle wasting and subduing free radicals produced as a result of hyperglycemia [64, 65].

Hyperlipidemia is caused by the excessive mobilization of fats from adipose tissue as a result of glucose underutilization in DM [66, 67]. Due to the unrestricted effects of lipolytic hormones on fat deposits, primarily as a result of the action of insulin, which is a risk factor for coronary heart disease, the levels of serum lipids are typically high in DM [68]. Furthermore, hyperglycemia is also associated with a rise in TC, TG, and LDL, as well as a decrease in HDL [69]. The reduction in TC, TG, and LDL compared to diabetic control and a significant increase in HDL levels of diabetic mice by the chloroform extract of *C. coggygria* could be attributed to the increased utilization of glucose that led to lipid peroxidation inhibition and controlling of lipolytic hormones. The liver enzyme markers such as AST, ALT, and ALP have still remained the standard for the assessment of hepatic toxicity and have been used as biomarkers of choice for decades. Tests of liver function may therefore prove useful in assessing especially the toxic effects of drugs on the liver. Increased gluconeogenesis, ketogenesis, and liver cell necrosis have been linked to the increased ALT, AST, and ALP levels in diabetic mice with insulin deficiency due to enzyme leakage from the liver cytosol into the bloodstream, suggesting that alloxan has a hepatotoxic effect [70]. In comparison to the diabetic mice not receiving treatment, the oral administration of chloroform extract at a concentration of 250 mg/kg remarkably reduced the activities of these enzymes.

Higher serum urea and creatinine levels in diabetic mice, two important indicators of renal failure, may be the result of a negative nitrogen balance brought on by the increased tissue proteolysis and decreased protein synthesis [71]. In the current investigation, it was discovered that diabetic mice fed with chloroform extract had considerably reduced the serum levels of urea and creatinine, indicating that the extract slowed the progression of renal impairment.

According to the reports, people with DM have higher levels of lipid peroxides and lower antioxidant enzyme activity [72]. Endogenous enzymatic antioxidants SOD, POD, and CAT protect cells from oxidative damage caused by harmful oxygen free radicals [73]. By converting superoxide anion into hydrogen peroxide, SOD reduces the toxicity of a subsequent reaction. CAT catalyzes the reduction of hydrogen peroxide and shields tissues against volatile hydroxyl radicals [74]. The most precarious of all the radicals, the hydroxyl radical, is produced by peroxidases when they interact with transitional metals [75]. Due to the direct free radical attack, high glucose levels in DM may cause lipid peroxidation, which can damage proteins and deactivate membrane-bound enzymes, resulting in induced-oxidative stress [76]. These results suggest that the extract's antioxidant capacity may be the driving force that underlies its antidiabetic effects.

The GC-MS analysis of the chloroform extract revealed numerous compounds having hypoglycemic and antioxidant potential which is comparable with the study of Naz et al. [77]. According to the study of Wang et al. [78], sulphuretin, dastin, fisetin, quercetin, taxifolin, gallic acid, 7-trihydroxyflavanone, myricetin, and disulphuretin are all present in *C. coggygria* extracts, which is in agreement with our research. The chloroform fraction from crushed and dried young leaves contained the largest amounts of total phenolic compounds (92.9%), tannins (83.4%), and flavonoids (3.5%) when compared to other fractions.

Numerous secondary metabolites, including flavonoids, phenols, alkaloids, tannins, saponins, and terpenoids, have been identified in plant extracts by phytochemical research. These might be the basis of the perceived antidiabetic activity. Due to their capacity to stimulate glycogenesis in the liver, release insulin from pancreatic *β*-cells, or inhibiting glucose absorption in the gut, the secondary metabolites that were found in *C. coggygria* extracts may be responsible for the antioxidant and observed glucose suppressive and antihyperglycemic activities [79–82]. Through a number of mechanisms, the utilization of these plants and phytoconstituents may prevent the onset of diabetic complications and regulate metabolic irregularities, which is in accordance with our previous research work [83, 84]. In order to integrate plant medicines with high therapeutic effects to treat diabetes, these results stimulate future investigations of the extracts and identify particular active molecules engaged in various biological functions.

## 5. Conclusion

In our present investigation, we have found out that there are many bioactive compounds in the plant extract mainly flavonoids, phenols, saponins, terpenes, glycosides, and steroids for pharmacological evaluations. From our studies, it is evident that these bioactive compounds from *C. coggygria* extract exerted potential mechanisms of action with speculating against antihyperglycemic and antioxidant activity. The *C. coggygria* extract possesses a stimulatory effect against antidiabetic activities in diabetic mice and showed a consistent effect in the restoration of the glucose levels, lipid profile, and other biochemical parameters supporting its traditional utilization. Further study needs to isolate, identify the active compounds, and target the possible mechanism of actions and combination of plant products with synthetic drugs.

## Figures and Tables

**Table 1:** Quantitative determination of phytochemicals.

S. no	Extract/fractions	TF (mg/g)	TP1 (mg/g)	TT (mg/g)	TS (mg/g)	TP2 (mg/g)
1	Methanolic extract	3.606 ± 0.13	3.829 ± 0.18	2.3935 ± 0.37	2.059 ± 0.46	0.5062 ± 0.34
2	n-Hexane	2.696 ± 0.21	3.075 ± 0.23	2.7445 ± 0.26	1.409 ± 0.61	0.1042 ± 0.27
3	Chloroform	3.470 ± 0.17	3.967 ± 0.32	2.5768 ± 0.52	2.799 ± 0.35	0.3334 ± 0.14
4	Ethyl acetate	3.292 ± 0.24	3.694 ± 0.21	2.8208 ± 0.29	4.357 ± 0.42	1.1594 ± 0.38
5	Aqua	2.316 ± 0.29	3.347 ± 0.34	2.7756 ± 0.32	3.910 ± 0.47	2.1499 ± 0.22

TF = total flavonoids; TP1 = total phenol; TT = total tannin; TS = total sugar; TP2 = total protein content. Data are expressed as mean ± SD, *n* = 3.

**Table 2:** *In vitro* antioxidant assays.

S. no	Extract/fraction	Conc. mg/ml	DPPH	% inhibition	H_2_O_2_ assay	% inhibition
1	Methanolic	0.1	0.0260 ± 0.003	70.5	0.0401 ± 0.015	49.4
2	n-Hexane	0.1	0.0324 ± 0.004	63.3	0.0711 ± 0.089	10.3
3	Chloroform	0.1	0.0125^∗^ ± 0.003	85.8	0.0207^∗^ ± 0.005	73.8
4	Ethyl acetate	0.1	0.0413 ± 0.003	53.2	0.0688 ± 0.007	13.2
5	Aqueous	0.1	0.0432 ± 0.004	51.1	0.0513 ± 0.035	35.3
6	Ascorbic acid (control)	0.1	0.0884 ± 0.003	93.2	0.0793 ± 0.017	86.2

Data are presented as mean ± SEM of each triplicate test.

**Table 3:** Effect of *C. coggygria* extracts on fasting blood glucose level in mice.

S. no	Treatments	Dose (mg/kg)	0 day	4 days	Blood glucose level in mg/dl 7 days	11 days	15 days
1	Normal saline	0.03 ml	100.8 ± 3.2	98.8 ± 2.5	96.8 ± 2.6	97.8 ± 3.1	95 ± 5.2
2	Diabetic control	0.03 ml	350.6 ± 3.6	367 ± 2.07	387 ± 3.9	409.8 ± 4.2	417 ± 4.3
3	Glibenclamide	10	348.2 ± 2.2	305^∗^ ± 2.03	258.8^∗^ ± 5.4	203.6^∗∗^ ± 3.9	181.6^∗∗^ ± 6.1
4	Methanolic extract	250	343 ± 1.9	330.8 ± 5.1	316.2 ± 5.04	308^∗^ ± 4.4	293^∗^ ± 4.8
5	n-Hexane	250	332.8 ± 3.4	321.2 ± 2.01	335.6 ± 3.1	344.2 ± 2.8	353.8 ± 3.2
6	Chloroform	250	342.8 ± 2.7	320.2^∗^ ± 1.8	289.8^∗^ ± 2.8	244.6^∗∗^ ± 3.01	214.6^∗∗^ ± 3.6
7	Ethyl acetate	250	317.6 ± 4.2	328.4 ± 1.7	341.2 ± 1.6	353.2 ± 1.08	366.2 ± 2.4
8	Aqua	250	330.4 ± 3.3	339 ± 1.3	348.4 ± 1.07	362.2 ± 2.7	375 ± 3.3

Values indicate mean ± SEM (*n* = 6). ^∗^*p* < 0.05 and ^∗∗^*p* < 0.01 compared with diabetic control values.

**Table 4:** Effect of *C. coggygria* extracts on body weight of mice.

S. no	Treatments	Dose (mg/kg)	0 day	4 days	Body weight (g) 7 days	11 days	15 days
1	Normal saline	0.03 ml	27.8 ± 0.42	27.8 ± 0.73	28.4 ± 0.91	29.3 ± 0.87	30.4 ± 0.72
2	Diabetic control	0.03 ml	26.6 ± 0.91	25.1 ± 0.51	24.3 ± 0.58	23.5 ± 0.82	23.1 ± 0.59
3	Glibenclamide	10	26.7 ± 1.1	27.1 ± 0.88	28.3^∗^ ± 0.47	31^∗^ ± 1.04	33.2^∗∗^ ± 0.74
4	Methanolic extract	250	28.3 ± 1.2	27.2 ± 1.02	27.1 ± 0.81	28.8 ± 0.92	30.8^∗^ ± 0.41
5	n-Hexane	250	26.5 ± 1.73	25.6 ± 1.1	25.1 ± 0.54	24.5 ± 0.78	23.7 ± 0.37
6	Chloroform	250	29.2 ± 0.86	30.1 ± 0.57	31.3^∗^ ± 1.03	31.9^∗^ ± 0.55	33.6^∗∗^ ± 0.45
7	Ethyl acetate	250	25.6 ± 0.77	24.9 ± 0.69	24.1 ± 0.83	23.6 ± 0.47	22.7 ± 0.49
8	Aqua	250	25 ± 0.91	24.4 ± 0.94	23.8 ± 0.71	23.5 ± 0.58	22.8 ± 0.38

Values indicate mean ± SEM (*n* = 6). ^∗^*p* < 0.05 and ^∗∗^*p* < 0.01 compared with diabetic control values.

**Table 5:** Effect of extracts on lipid profile.

S. no	Treatments	Dose (mg/kg)	TC (mg/dl)	TG (mg/dl)	LDL (mg/dl)	HDL (mg/dl)
1	Normal saline	0.03 ml	122.3 ± 1.7	156.3 ± 1.6	73.3 ± 2.2	36.5 ± 3.2
2	Diabetic control	0.03 ml	152.6 ± 2.3	183.2 ± 0.76	132.3 ± 1.4	34.3 ± 1.9
3	Glibenclamide	10	126.1^∗∗^ ± 0.97	159.5^∗∗^ ± 1.3	78.3^∗∗^ ± 2.2	46.3^∗∗^ ± 2.7
4	Methanolic extract	250	134.3^∗^ ± 2.4	175.2^∗^ ± 2.8	103.3^∗^ ± 1.8	42.2^∗^ ± 2.1
5	n-Hexane	250	146.1 ± 0.88	183.2 ± 1.07	114.3 ± 3.2	35.4.1 ± 2.9
6	Chloroform	250	131.2^∗∗^ ± 1.9	161.5^∗∗^ ± 2.5	79.2^∗∗^ ± 2.3	43.4^∗∗^ ± 1.8
7	Ethyl acetate	250	147.3 ± 2.01	180.3 ± 3.4	103.5 ± 3.2	40.7 ± 2.7
8	Aqua	250	146.2 ± 1.4	182.5 ± 0.98	118.5 ± 0.91	40.2 ± 0.78

Values indicate mean ± SEM (*n* = 6). ^∗^*p* < 0.05 and ^∗∗^*p* < 0.01 compared with diabetic control values.

**Table 6:** Effect of extracts on serum biochemical profile.

S. no	Treatments	Dose (mg/kg)	ALT (U/I)	AST (U/I)	ALP (U/I)	Creatinine (mg/dl)	Urea (mg/dl)
1	Normal saline	0.03 ml	10.5 ± 0.77	15.4 ± 0.91	206.6 ± 0.44	1.46 ± 0.56	24.12 ± 1.21
2	Diabetic control	0.03 ml	35.2 ± 0.63	34.4 ± 0.71	345.4 ± 0.98	3.26 ± 1.2	39.13 ± 2.1
3	Glibenclamide	10	15.5^∗∗^ ± 0.46	21.4^∗∗^ ± 0.61	215.3^∗∗^ ± 0.42	1.16^∗∗^ ± 0.36	28.21^∗∗^ ± 0.94
4	Methanolic extract	250	26.1 ± 0.89	25.2 ± 2.1	245.3 ± 1.8	2.36 ± 1.4	35.4 ± 1.3
5	n-Hexane	250	31.1 ± 1.1	30.1 ± 0.34	298.2 ± 0.52	3.09 ± 0.77	38.8 ± 1.7
6	Chloroform	250	23.4^∗^ ± 1.7	23.1^∗^ ± 1.2	238.3^∗^ ± 0.65	2.16^∗^ ± 0.76	30.7^∗∗^ ± 2.2
7	Ethyl acetate	250	30.8 ± 0.89	28.2 ± 1.3	293.06 ± 1.4	3.14 ± 0.94	37.6 ± 1.6
8	Aqua	250	32.1 ± 0.72	30.3 ± 0.87	296.2 ± 1.5	3.25 ± 1.2	37.9 ± 1.03

Values indicate mean ± SEM (*n* = 6). ^∗^*p* < 0.05 and ^∗∗^*p* < 0.01 compared with diabetic control values.

**Table 7:** Antioxidant enzyme activities in normal and diabetic mice.

S. no	Treatments	Dose (mg/kg)	SOD (U/mg)	POD (U/mg)	Catalase (U/mg)
1	Normal saline	0.03 ml	6.33 ± 0.12	4.71 ± 0.52	5.92 ± 1.2
2	Diabetic control	0.03 ml	4.56 ± 0.23	1.98 ± 0.78	2.31 ± 0.96
3	Glibenclamide	10	5.84^∗∗^ ± 0.31	3.93^∗∗^ ± 0.64	4.77^∗∗^ ± 0.83
4	Methanolic extract	250	5.01^∗^ ± 0.35	2.61 ± 0.36	2.97^∗^ ± 0.62
5	n-Hexane	250	3.77 ± 0.67	2.03 ± 0.46	2.22 ± 1.3
6	Chloroform	250	5.15^∗^ ± 0.73	2.77^∗^ ± 0.82	3.01^∗^ ± 1.1
7	Ethyl acetate	250	3.65 ± 0.45	2.05 ± 0.71	2.36 ± 0.79
8	Aqua	250	3.54 ± 0.27	2.03 ± 0.38	2.18 ± 0.44

Values indicate mean ± SEM (*n* = 6). ^∗^*p* < 0.05 and ^∗∗^*p* < 0.01 compared with diabetic control values.

**Table 8:** Thrombolytic activity of extracts of *C. coggygria.*

S. no	Treatments	Conc. mg/ml	Thrombolytic activity	% inhibition
1	Methanolic extract	1	0.0748^∗^ ± 1.2	69.93
2	n-Hexane	1	0.2809 ± 2.02	40.15
3	Chloroform	1	0.0284^∗∗^ ± 0.56	84.97
4	Ethyl acetate	1	0.5881 ± 0.83	16.58
5	Aqua	1	0.5211 ± 1.04	26.08
6	Acetylsalicylic acid	0.10	0.705^∗∗^ ± 0.86	89

Data are expressed as mean ± SD (*n* = 3), ^∗^*p* < 0.05, and ^∗∗^*p* < 0.01.

**Table 9:** FTIR profile of *C. coggygria*.

S. no	Peak (wave number cm^−1^)	Reference article (wave number cm^−1^)	Bond	Type of vibration	Functional group assignment
1	3284.49	3400-3200	O-H	Stretch	Alcohol/phenol
2	2919.29	2935-2915	C-H	Stretch	Alkanes
3	1600	1650-1600	C=O	Stretch	Ketone compound
4	1477.85	1510-1450	C=C	Stretch	Aromatic compound
5	1260.39	1340-1250	C-N	Stretch	Primary amine
6	866.69	995-850	C-Cl	Stretch	Aliphatic chlorocompounds

**Table 10:** GC-MS analysis of *C. coggygria*.

S. no	Compound name	Area %	RT	Molecular formula	MW
1	3-Methylpentane	93.49	0.65	C_6_H_14_	86
2	Propane, 1,1-dimethoxy-2-methylundecane	0.66	1.14	C_6_H_14_O_2_	118
3	1,1*-*Dimethoxy*-*2*-*propanone	0.66	1.14	C_5_H_10_O_3_	118
4	2-Oxiranecarboxylic acid	0.35	1.90	C_9_H_16_O	204
5	Carotene	0.24	2.20	C_41_H_58_O	566
6	9,12,15-Octadecatrienoic acid	0.18	3.65	C_27_H_52_O_4_Si_2_	496
7	Strychane	0.10	5.30	C_21_H_26_N_2_O_2_	338
8	Glucobrassicin	0.10	5.30	C_16_H_20_N_2_O_9_S_2_	448
9	9-Octadecenoic acid	0.09	4.15	C_57_H_104_O_6_	884
10	à-l-Galactopyranoside	0.09	4.15	C_14_H_29_BO_5_Si	316
11	2(1H)Isoquinolinecarboximidamide,3,4-dihydro-	0.23	6.58	C_10_H_13_N_3_	175
12	Dasycarpidan-1-methanol	0.26	6.91	C_20_H_26_N_2_O_2_	326
13	Milbemycin b	0.07	8.09	C_33_H_46_ClNO_7_	603
14	Hexadecanoic acid	0.57	9.00	C_36_H_56_O_6_	584
15	Curan-17-oic acid, 19,20-dihydroxy-methyl ester	0.07	10.08	C_20_H_26_N_2_O_4_	358
16	3-Phenylthioacrylic acid	0.11	11.20	C_16_H_11_NO_2_S	281
17	2-Bromotetradecanoic acid	0.55	12.26	C_14_H_27_BrO_2_	306
18	Oleic acid	0.55	12.26	C_18_H_34_O_2_	282
19	2-Myristynoyl pantetheine	0.95	13.51	C_25_H_44_N_2_O_5_S	484
20	2-Naphthalenemethanol	1.24	14.34	C_15_H_26_O	222
21	3-O-Methyl-d-glucose	8.64	16.52	C_7_H_14_O_6_	194
22	Pentadecanoic acid	3.22	18.31	C_17_H_34_O_2_	270
23	l-(+)-Ascorbic acid 2,6-dihexadecanoate	7.63	18.66	C_38_H_68_O_8_	652
24	Docosanoic acid	0.34	20.94	C_69_H_134_O_6_	1058
25	Hexa-t-butylselenatrisiletane	5.82	20.94	C_24_H_54_SeSi_3_	506
26	Cholestan-3-one, cyclic 1,2-ethanediyl aetal	0.51	21.23	C_29_H_50_O_2_	430
27	1,2-Benzenedicarboxylic acid	5.71	22.33	C_24_H_38_O_4_	390
28	Hexasiloxane	0.10	24.85	C_12_H_38_O_5_Si_6_	430
29	Tocopherol	22.34	25.91	C_29_H_50_O_2_	430
30	2-Methoxy-4-vinylphenol	0.92	9.27	C_9_H_10_O_2_	150
31	Stigmasterol	22.34	25.91	C_29_H_50_O	414
32	1-Monolinoleoylglycerol trimethylsilyl ether	0.06	27.09	C_27_H_54_O_4_Si_2_	498
33	Octasiloxane	0.68	29.11	C_16_H_50_O_7_Si_8_	578
34	n-Hexadecanoic acid	7.63	18.66	C_16_H_32_O_2_	256
35	Pregnane-3,20-dion	10.24	18.21	C_32_H_62_N_2_O_5_Si_3_	638
36	*β*-Sitosterol	1.38	20.30	C_29_H_50_O	414

RT = retention time; MW = molecular weight.

**Table 11:** Identified antidiabetic compounds from chloroform fraction using GC-MS.

S. no	Compounds	Nature of compound	Reference
1	Dasycarpidan-1-methanol acetate	Steroid	[49]
2	Hexadecanoic acid	Ester	[50]
3	Stigmasterol	Steroid	[51, 52]
4	1-Monolinoleoylglycerol trimethylsilyl ether	Steroid	[53]
5	*β*-Sitosterol	Steroid	[54]
6	Oleic acid	Ester	[55]
7	3-O-Methyl-d-glucose	Sugar	[56]
8	Docosanoic acid	Ester	[57]
9	Tocopherol	Vitamin	[58]
10	2-Methoxy-4-vinylphenol	Phenol	[59]

## Data Availability

Source data for tables and figures are provided in the manuscript.
